# The mitochondrial K-ATP channel opener diazoxide upregulates STIM1 and Orai1 via ROS and the MAPK pathway in adult rat cardiomyocytes

**DOI:** 10.1186/s13578-020-00460-w

**Published:** 2020-08-13

**Authors:** Joice T. Gavali, Elba D. Carrillo, María C. García, Jorge A. Sánchez

**Affiliations:** Departamento de Farmacología, Centro de Investigación y de Estudios Avanzados del IPN, Av. Instituto Politécnico Nacional 2508, 07360 Ciudad de México, CDMX Mexico

**Keywords:** Diazoxide, NFkB, c-Fos, ROS, STIM, Orai, SOCE, Mitochondrial K-ATP channels, Cardiomyocytes

## Abstract

**Background:**

Openers of mitochondrial adenosine triphosphate-dependent potassium (mKATP) channels like diazoxide increase reactive oxygen species (ROS) production in cardiac cells and reduce Ca^2+^ elevations produced by ischemia–reperfusion, protecting the heart from damage. In this study we tested the hypothesis that opening mKATP channels regulates expression of the major components of store-operated Ca^2+^ entry (SOCE) STIM1 and Orai1.

**Results:**

Quantitative reverse transcriptase polymerase chain reaction (qRT-PCR) and western blot experiments showed that diazoxide increased expression of STIM1 and Orai1 at the mRNA and protein levels, respectively, in adult rat cardiomyocytes. Immunofluorescence analyses revealed that diazoxide also disrupted the striated distribution pattern of STIM1. These effects were prevented by the ROS scavenger *N*-acetyl cysteine (NAC), the mKATP channel antagonist 5-hydroxydecanoate (5-HD), or the protein synthesis inhibitor cycloheximide (CHX). Confocal microscopy revealed that diazoxide also led to nuclear translocation of the transcription factors c-Fos and NFκB, which was also blocked by NAC or 5-HD. Finally, the MAPK pathway inhibitor UO126 attenuated diazoxide-induced upregulation of STIM1 and Orai1 expression.

**Conclusions:**

Our results suggest that opening mitochondrial potassium ATP channels with diazoxide upregulates the expression of STIM1 and Orai1 by de novo synthesis by a mechanism that involves NFkB, c-Fos, and ROS via MAPK/ERK signaling.

## Introduction

Voltage and store-operated Ca^2+^ channels are the major routes of Ca^2+^ influx in mammalian cells [1]. In many metazoan cells stimulation of store-operated Ca^2+^ entry (SOCE) is required to refill and maintain the endoplasmic reticulum Ca^2+^ content [1]. In addition, contraction and relaxation of cardiomyocytes depend on the regulation of cytosolic Ca^2+^ levels via the activities of voltage-dependent Ca^2+^ channels, pumps, and exchangers but the role of SOCE in cardiomyocyte function is less clear. Notwithstanding, cardiomyocytes have been demonstrated to express both components of SOCE, namely the endo-sarcoplasmic reticulum Ca^2+^ sensor, stromal interaction molecule 1 (STIM1) [2–7] and its associated Ca^2+^ channel, Orai1 [2–5, 8].

While the physiological role of SOCE in the adult heart continues to be debated, SOCE is thought to be involved in development because high expression levels of STIM1 are seen in neonatal cardiomyocytes [9, 10]. Additionally, SOCE has been implicated in pathological conditions of the heart, such as cardiac hypertrophy, a disorder that leads to upregulation of STIM1 [3, 9, 10]. Whether and how expression of STIM1 and Orai1 may be altered in other pathophysiological states remain largely unexplored.

Ischemic preconditioning is an endogenous phenomenon whereby brief periods of ischemia and reperfusion result in subsequent protection from acute myocardial infarction [11]. It can be induced pharmacologically with mKATP channel openers, such as diazoxide (Dzx), leading to increases in mitochondrial reactive oxygen species (ROS) production [12–14]. The protein components of mKATP channels have been recently described and most importantly, loss of mKATP channels prevents Dzx-induced cardioprotection in ischemia–reperfusion experiments indicating that mKATP channels are the targets of Dzx during pharmacological preconditioning [14]. Incubation with Dzx also reduces the amplitude of Ca^2+^ currents through Cav1.2 channels and the expression of the principal subunit of these channels in adult hearts [15] and promotes inactivation of SOCE in adult cardiomyocytes [16]. Interestingly, a reciprocal interaction between voltage-activated Cav1.2 channels and store-operated Ca^2+^ channels, mediated by STIM1, has been reported [17, 18].

In this study, we examined the effects of Dzx on the transcription and protein expression of STIM1 and Orail, and on the intracellular localization of the transcription factors c-Fos and NFκB. We used western blot and quantitative reverse transcriptase polymerase chain reaction (qRT-PCR) experiments to quantify protein and mRNA transcript levels in presence of various drugs aimed at probing the mechanisms involved. We examined whether ROS and the MAPK (mitogen-activated protein kinase)/ERK pathway are involved in the upregulation of Orai1 and STIM1 proteins by Dzx, and tested the possibility that changes in the expression of SOCE components depend on c-Fos and NF-κB transcription factors.

## Materials and methods

### Animals

Adult male (250–300 g) Wistar rats were used. Our experimental protocols were approved by the Division of Laboratory Animal Units, Cinvestav-IPN, and were in compliance with federal law and *Consejo Nacional de Ciencia y Tecnología* regulations.

### Isolation of hearts

In preparation for heart extraction, each rat was anesthetized with 50 mg/kg sodium pentobarbital and given 500 U/kg heparin sodium solution (both administered by intraperitoneal injection). When the rat was completely unresponsive to stimulation, its heart was excised rapidly, arrested in modified Krebs–Henseleit buffer (containing, in mM: 117.8 NaCl, 1.2 NaH_2_PO_4_, 6.0 KCl, 24.3 NaHCO_3_, 1.2 MgSO_4_, 0.027 EDTA, 5.1 glucose and 1.6 CaCl_2_), gassed with 95% O_2_/5% CO_2_ at pH 7.4, and perfused in a Langendorff apparatus with an aortic cannula. Unless otherwise stated, all chemicals and materials were purchased from Sigma-Aldrich (St. Louis, MO).

Isolated hearts in the control group were perfused with Krebs–Henseleit buffer for 90 min. Those in the Dzx-treated (Tocris, Bristol, UK) group were perfused in Krebs–Henseleit buffer containing 100 μM Dzx for 90 min. Hearts in the NAC-Dzx group were first exposed to Krebs–Henseleit buffer with 4 mM *N*-acetyl cysteine (NAC), a reactive oxygen species (ROS) scavenger, for 15 min. Thereafter, the perfusion buffer was supplemented with 100 μM Dzx for 90 min. Hearts in the NAC group were first perfused with Krebs–Henseleit buffer for 90 min, to which NAC (4 mM) was added. For experiments in which the mKATP channel antagonist 5-hydroxydecanoate (5-HD) was used, 100 μM 5-HD was applied as described above for NAC.

### Isolation of ventricular myocytes

Excised hearts were perfused for 5 min at 37 °C with Ca^2+^-free Tyrode’s solution containing (in mM): 136 NaCl, 5.4 KCl, 1 MgCl_2_, 10 HEPES, and 11 glucose. Hearts were recirculated for 60 min with Tyrode’s solution supplemented with 70-U/mL type II collagenase (Worthington, Lakewood, NJ) and 0.5-mg/100 mL type XIV protease. Ventricles were minced and shaken 2–3 times at 45 rpm for 7 min in the same solution. The dislodged cells were filtered through a 100-μm nylon cell strainer (BD Falcon) and centrifuged at 28×*g* for 2 min. The pellet was resuspended in Tyrode’s solution with 1% bovine serum albumin (BSA).

### Cardiomyocyte treatments

Resuspended pellets were maintained for 90 min in Tyrode solution plus 1% BSA in control experiments, or in an identical solution containing Dzx (100 μM), 5-HD (100 μM), or NAC (2 mM or 4 mM). To test the involvement of the MAPK pathway, we used 5 μM 1,4-diamino-2,3-dicyano-1,4-bis(methylthio) butadiene (UO126), a selective noncompetitive inhibitor of the MAPK kinases, MEK1 and MEK2. Cardiomyocytes were preincubated for 1 h in Tyrode solution containing UO126 and then Dzx was added to this solution and cardiomyocytes were incubated for additional 90 min. Cardiomyocytes were exposed to 10 μM cycloheximide (CHX), a selective inhibitor of protein synthesis, for 30 min and then incubated for 90 min in the same solution with 100 μM Dzx added. To test the involvement of ROS, we added 100 μM H_2_O_2_ to Tyrode solution for 10 min and then cardiomyocytes were incubated for additional 90 min in H_2_O_2_-free Tyrode solution.

All drugs were removed by washing three times with Tyrode’s solution containing BSA (1 mg/mL) and 1-mM CaCl_2_. Thereafter, cells were centrifuged at 28×*g* for 2 min, and total proteins were extracted for western blot analysis.

### Membrane fractionation and western blotting

To obtain the membrane fraction, heart tissue was homogenized in ice-cold lysis buffer containing (in mM) 20 Tris (pH 7.4), 5.0 EDTA, 250 sucrose, 1.0 phenylmethanesulfonylfluoride, and 2.5% protease inhibitor mixture, as described elsewhere [19]. Tissue homogenates (20% w/v) were centrifuged at 1000×*g* for 10 min to remove nuclei and debris, and the supernatant was ultracentrifuged at 110,000×*g* for 75 min at 4 °C to pellet the crude membrane fraction (sarcolemmal and microsomal subfractions). The resulting pellet was resuspended in solubilization buffer containing (in mM) 50 Tris (pH 7.4), 100 NaCl, 50 LiCl, 5 EDTA, 0.5% (v/v) Triton X-100, 0.5% (w/v) sodium deoxycholate, 0.05% (w/v) sodium dodecyl sulfate (SDS), and 0.02% (w/v) sodium azide. After incubation for 30 min on ice, the remaining insoluble material was collected by centrifugation (14,000×*g*, 10 min, 4 °C). Protein content of the supernatant (particulate membrane fraction) was measured with the Bradford method.

Dissociated myocytes used for western blotting were resuspended in lysis buffer containing 20 mM Tris (pH 7.5), 100 mM NaCl, 1% Triton X-100, and protease inhibitors. Lysis was achieved by vortexing every 10 min, for 60 min, at 4 °C, followed by five cycles of sonication. Samples were centrifuged at 13,000×*g* for 10 min at 4 °C and the soluble fraction was used for western blots. Protein content was measured with Bradford assays.

Whole-membrane fractions from ventricles or total fraction samples from isolated cardiomyocytes (50–60 μg) were subjected to 10% SDS–polyacrylamide gel electrophoresis (180 V, 120 min). The resultant protein bands were transferred onto nitrocellulose membranes, blocked with 4.5% nonfat dried milk in PBS, and probed with anti-STIM1 monoclonal antibody (1:1000; Abcam, Cambridge, UK), anti-Orai1 polyclonal antibody (1:3000; Abcam, Cambridge, UK), anti–phosphorylated-44/42 MAPK (pERK1/2) polyclonal antibody (1:1000; Cell Signaling Technology, Danvers, MA, USA) and anti–ERK1/2-44/42 MAPK (ERK1/2) monoclonal antibody (1:500; Santa Cruz Biotechnology Inc., Dallas, TX, USA). This antibody recognizes two bands of total ERK with molecular weights of 44 kDa (ERK1) and 42 kDa (ERK2), being the most abundant one the band of lower molecular weight. Finally, as loading controls we used anti-actin monoclonal antibody (1:2000; Sigma Aldrich, St. Louis, MO, USA) and anti-GAPDH monoclonal antibody (1:15,000; Sigma Aldrich, St. Louis, MO, USA) in PBS for 12–14 h at 4 °C. After incubation, membranes were rinsed three times with PBS-Tween20 (0.1%), incubated for 1 h with anti-rabbit (1:50,000) or anti-mouse (1:75,000) horseradish peroxidase-conjugated secondary antibody (Invitrogen, Carlsbad, CA, USA) in PBS and rinsed with PBS-Tween 20 (0.1%). Antibody labeling was detected with Immobilon western reagent (Millipore Co, Billerica, MA, USA) according to the manufacturer’s instructions.

### Immunofluorescence

Freshly isolated adult rat cardiomyocytes were suspended in Tyrode’s solution containing 1 mM CaCl_2_ plus 5% fetal bovine serum (Thermofisher Scientific, Waltham, MA, USA), plated onto laminin-treated slides and allowed to settle for 2 h at 37 °C. Attached cardiomyocytes were then subjected to the aforementioned drug treatments. All drugs were removed by washing three times with Tyrode’s solution containing 1 mM CaCl_2_. Immediately after the treatments, cells were fixed with 4% paraformaldehyde in PBS for 15 min at 4 °C. The fixed cells were washed three times with PBS, and then permeabilized and blocked in PBS containing 0.3% Triton X-100 and 5% donkey serum for 1 h at room temperature. Cells were then incubated overnight at 4 °C with primary antibodies: monoclonal anti-STIM1 (1:50; Abcam, Cambridge, UK), polyclonal anti-Orai1 (1:50; Thermofisher Scientific, Waltham, MA, USA), monoclonal anti c-Fos (1:50, Santa Cruz, CA, USA), monoclonal anti NFκBp65 (1:50; Santa Cruz, CA, USA) in PBS containing 0.3% Triton X-100 and 0.5% BSA. After washing three times in PBS, cells were incubated for 1 h at room temperature with secondary antibodies: Alexa Fluor 555-conjugated donkey anti-rabbit (1:200; Thermofisher Scientific, Waltham, MA, USA) for Stim1 and Alexa Fluor 488-conjugated donkey anti-mouse (1:200; Thermofisher Scientific, Waltham, MA, USA) for Orai1 and then washed three times in PBS. Thereafter, cells were incubated for 10 min at room temperature with Hoechst nuclear stain (1:1000; Invitrogen, Carlsbad, CA, USA). Negative controls were processed without primary antibodies.

Labeling was visualized under a laser scanning confocal microscope (Leica, Wetzlar, Germany, model TCS-SP8) with argon (488 nm) and helium/neon (543 nm) lasers used with an optimized pinhole diameter. Confocal images were obtained as z-stacks of single optical sections, which were superimposed as a single image in Leica LAS AF 2.6.0 build 7268 software. Immunofluorescence was quantified in ImageJ 1.44 p (NIH, Bethesda, MD, USA) after the images were threshold adjusted (pixel value used to find edges of immunolabelling closed regions) at an intensity of twice the mean intensity and three (for STIM1) or five (for Orai1) times the standard deviation. The threshold area was outlined under particle analysis (size 0-infinity; circularity 0–1).

### qRT-PCR

RNA was isolated from cardiomyocytes with RLT buffer and a RNeasy Mini Kit (Qiagen, Hilden, Germany), and cDNAs were synthesized with a Taqman reverse transcription kit (Thermofischer, Waltham, MA, USA). Transcript levels were determined in TaqMan assays (Rn02397170_m1, Rn01506496_m1; Applied Biosystems, Foster City, CA, USA) and an iCycler iQ machine (Bio-Rad, Hercules, CA) with TaqMan Gene Expression Master Mix (Thermofischer, Waltham, MA, USA). mRNA expression was assessed relative to the ribosomal RNA 18S (Hs999999_s1, Applied Biosystems, Foster City, CA, USA), as recommended by the manufacturer. Relative changes in expression were calculated by the 2^−ΔΔCT^ method [20].

### Data analysis

The data, which are expressed as means ± standard errors of the mean (SEMs), were tested for normal distribution, analyzed with independent t-tests (when two groups were compared) or with analyses of variance (ANOVAs) followed by multiple comparison Dunnett’s tests (each treatment group vs. control group). A significance criterion of p < 0.05 was used.

## Results

### Upregulation of STIM1 and Orai1by Dzx

**Fig. 1:**
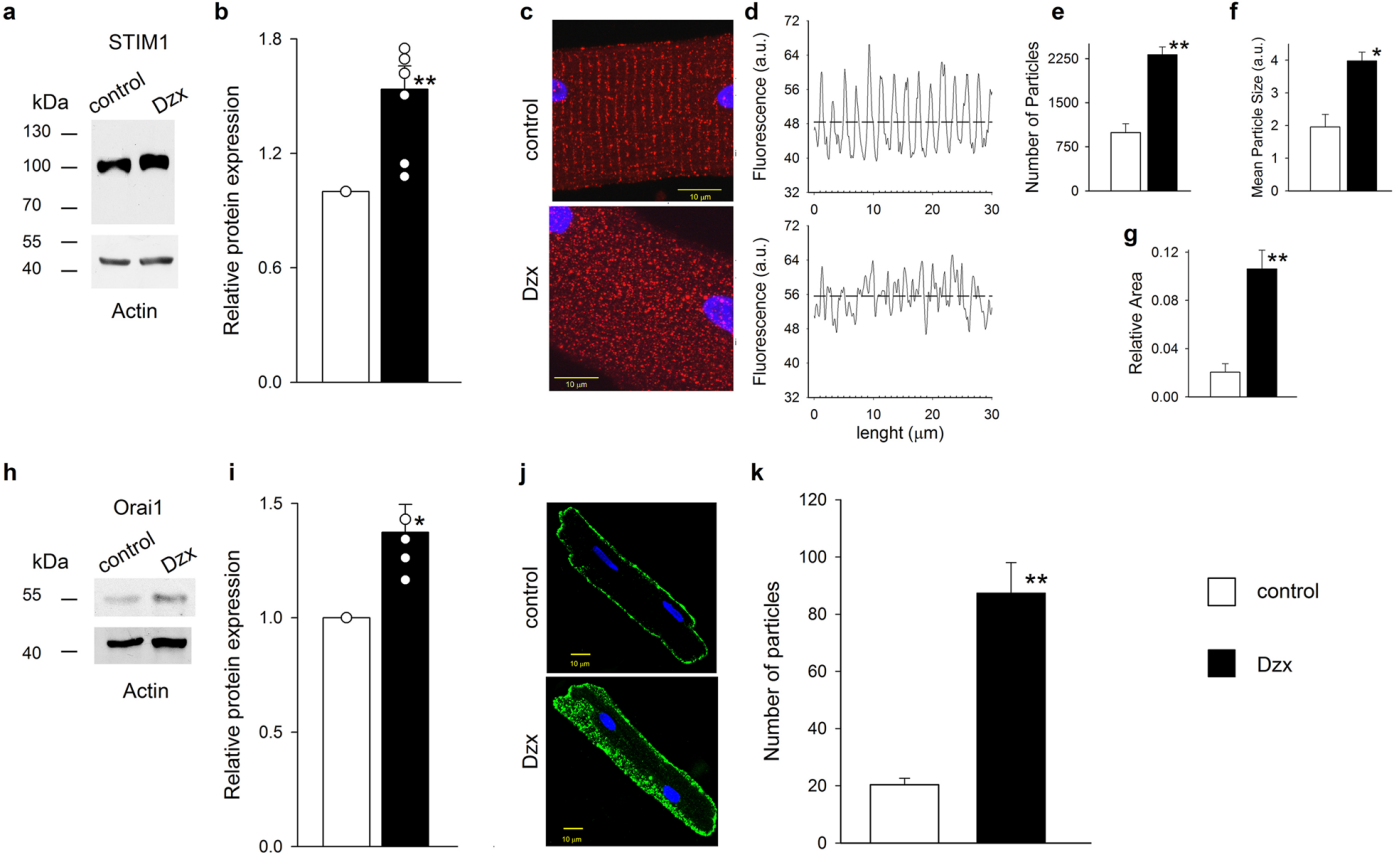
Dzx increases STIM1 and Orai1 expression. **a** A representative STIM1 western blot of whole membrane fractions from control and Dzx-treated hearts with actin as a loading control. **b** Means (± SEM, n = 7) of normalized densities of STIM1 bands. Open circles represent individual determinations. **c** Confocal microscopy images of cardiomyocytes under control conditions and after Dzx-treatment. Images show localization of STIM1 (red) with DAPI-labeled nuclei (blue). Calibration bar, 10 μm. **d** Mean fluorescence intensity along the longitudinal axis of the cardiomyocytes shown in **c**, the dotted lines indicate averages of all values. **e**–**g** Particle analysis of STIM1 in cardiomyocytes under the same conditions as in **c**. Mean numbers of particles (**e**), mean particle size (**f**), and ratios of total particle area and cell area (**g**) (± SEM, n = 5–10). Empty bars, control; filled bars, Dzx. **h** Representative Orai1 western blot of whole membrane fractions from control and Dzx-treated hearts, with an actin loading control. **i** Mean normalized densities of Orai1 bands (± SEM, n = 4). Open circles represent individual determinations. **j** Representative immunofluorescence images of cardiomyocytes showing distributions of Orai1 under the indicated experimental conditions. **k** Mean (± SEM, n = 6–10) number of particles of Orai1 from confocal images of cardiomyocytes under experimental conditions as in **j**. *p  < 0.05, **p < 0.01

Western blots showed that the abundances of the STIM1 (Fig. 1a, b) and Orai1 (Fig. 1h, i) proteins were significantly increased following Dzx incubation. Immunofluorescence imaging revealed that Dzx also changed the distribution of STIM1 in cardiomyocytes relative to that seen under control conditions (Fig. 1c–g). In control cardiomyocytes, STIM1 had a characteristic cross-striated distribution, with STIM1 organized into puncta and linear structures along the Z-disk (Fig. 1c). This distribution was analyzed in Fig. 1d which shows mean fluorescence intensity values along the longitudinal axis, calculated as the average fluorescence intensity along the straight line orthogonal to the longitudinal myocyte axis passing through a given point. In control experiments, fluorescence points were aligned leading to regularly spaced peaks of mean fluorescence intensity since STIM1 is located mostly along the Z lines. In diazoxide-treated cardiomyocytes the mean fluorescence intensity did not show periodic behavior along that axis, as the spatial organization of fluorescence was largely absent and the pattern of distribution was severely disrupted (Fig. 1d). Consistent with western blot results, there was also an increase in overall fluorescence values (Fig. 1d, dotted lines) and, the numbers of particles and their mean size increased sharply (Fig. 1e, f), leading to an increase in the ratio of total particle area and cell area (Fig. 1g). In adult cardiomyocytes Orai1 does not follow the striated pattern of STIM1 distribution [7] as we also observed in control experiments. In Dzx-treated cardiomyocytes we found that Orai1 also was expressed along the surface membrane albeit at much greater levels (Fig. 1j) and the main effect of Dzx was an increase in the number of particles associated with Orai1 fluorescence (Fig. 1k).

### Involvement of mKATP channels and ROS in Dzx-induced upregulation of STIM1 and Orai1

**Fig. 2:**
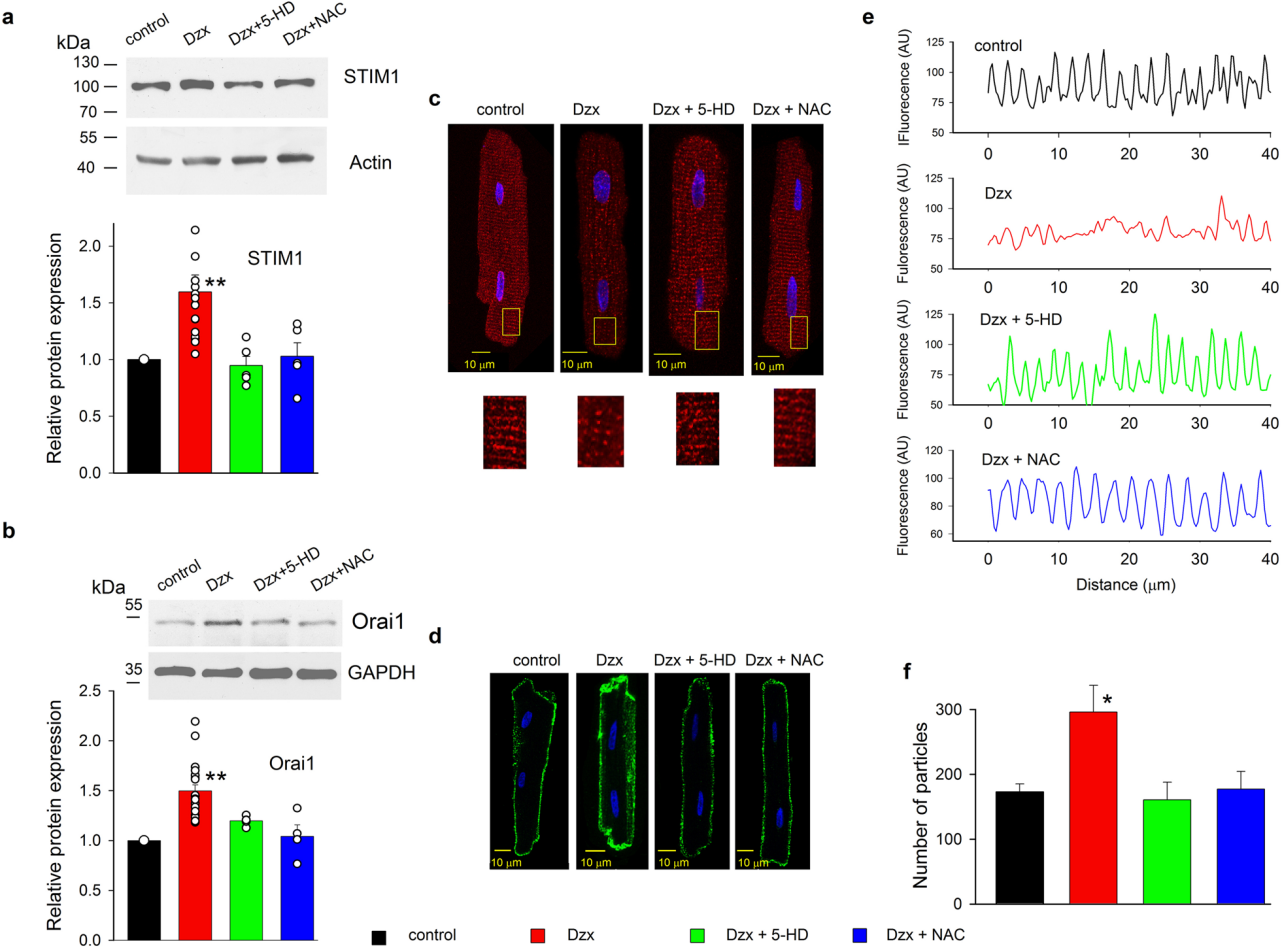
Upregulation and distribution changes of STIM1 and Orai1 proteins by Dzx depend on ROS and the opening of mKATP channels. **a** Representative western blots of STIM1 from whole membrane fractions of ventricles. The graph shows mean (± SEM, n = 5–15) normalized densities of STIM1 bands under the indicated experimental conditions. Open circles represent single determinations. **b** Representative western blots of Orai1 from whole membrane fractions of ventricles under experimental conditions as in **a**. The graph shows mean (± SEM, n = 4–23) normalized densities of Orai1 bands under the indicated experimental conditions. Actin was used as a loading control in all cases. Open circles represent single determinations. **c** Upper panels, representative confocal images of cardiomyocytes under the indicated experimental conditions. Lower panels, enlargements of yellow boxed areas. Images show localization of STIM1 (red) with DAPI-labeled nuclei (blue). **d** Representative confocal images of cardiomyocytes under the indicated experimental conditions. Images show localization of Orai1 (green) with DAPI-labeled nuclei (blue). Calibration bars, 10 μm in all cases. **e** Plot fluorescence intensity profiles along the longitudinal axis of the cardiomyocytes shown in **c**. **f** Particle analysis of cardiomyocytes under the same conditions as in **d**. Mean particle counts (± SEM, n = 5–7) are shown. *p < 0.05, **p < 0.01

We have previously reported that Dzx-incubation protects the heart from ischemic-reperfusion damage through the increase in ROS production [15, 16] and recent evidence indicates that the increase in the rate of ROS production produced by Dzx is prevented in the absence of mKATP channels [14]. Therefore, the mKATP channel blocker 5-HD and the ROS scavenger NAC were used to assess whether Dzx-induced increases in STIM1 and Orai1 protein abundance also depend on ROS and on the opening of mKATP channels. We found that STIM1 and Orai1 protein abundances remained unchanged by Dzx-treated cardiomyocytes that were preincubated with 5-HD or NAC (Fig. 2a, b). Further support for a role of ROS on increased expression of STIM1 was obtained by the use of H_2_O_2_. The relative protein abundance of STIM1 increased after incubation in H_2_O_2_ (Additional file 1: Figure S1).

Immunofluorescence imaging analysis demonstrated that disruption of STIM1 distribution by Dzx was also blocked when 5-HD or NAC were added to Dzx-containing solution (Fig. 2c). Plots of mean fluorescence intensity values of the images of STIM1 immunolabelling confirmed the role of ROS and mKATP channels in the distribution changes of STIM1 by Dzx (Fig. 2e). Meanwhile, Dzx-induced increases in the surface expression of Orai1 (Fig. 2d) and the number of Orai1 particles (Fig. 2f) were abrogated completely by the mKATP channel blocker 5-HD or by the ROS scavenger NAC.

### Increased STIM1 and Orai1 protein levels produced by de novo protein synthesis

**Fig. 3:**
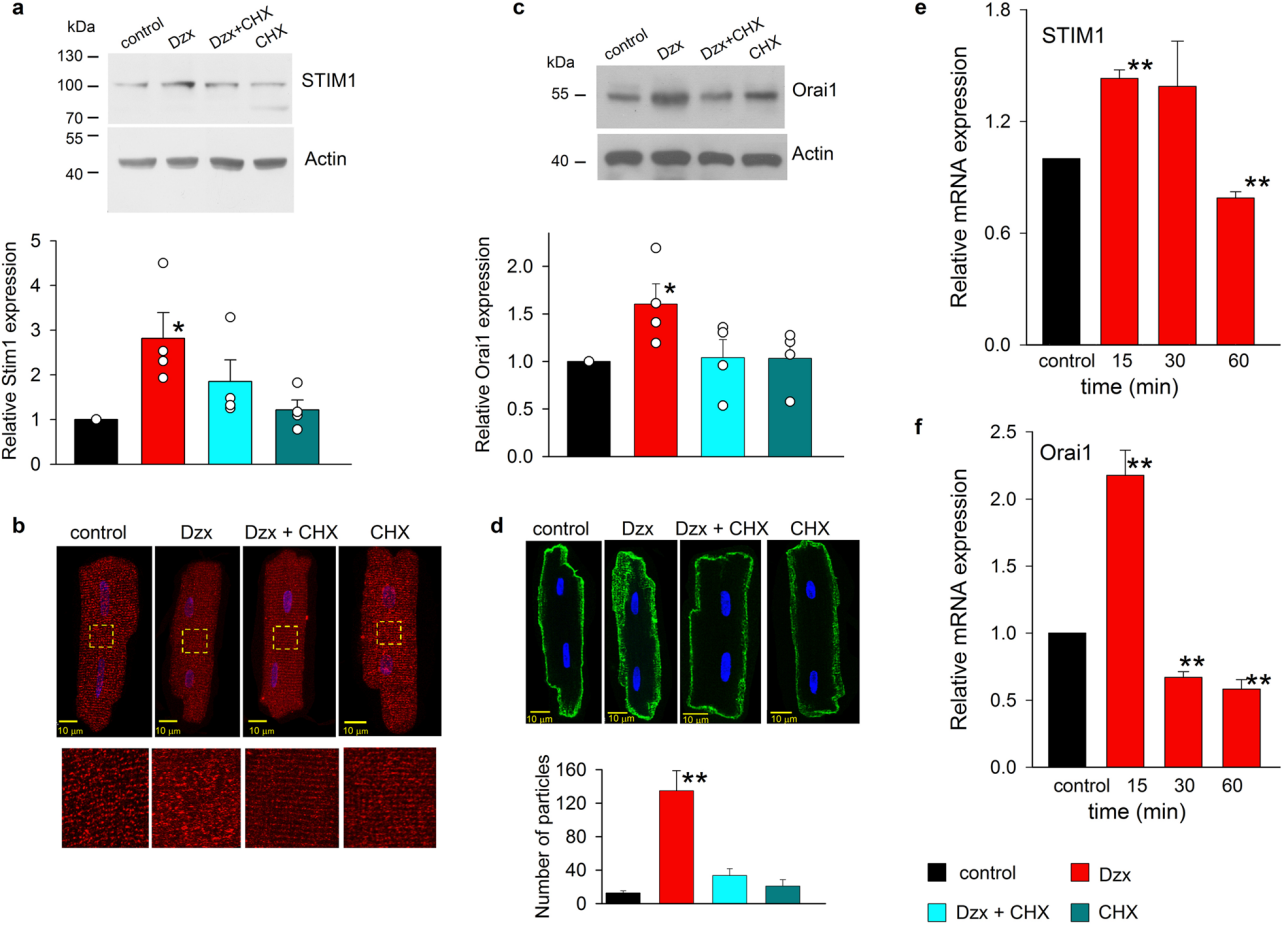
Dzx effects on STIM1 and Orai1 expression and localization depend on synthesis *de novo* as revealed by the protein synthesis inhibitor CHX and mRNA determinations. **a** Representative western blots of STIM1 from cardiomyocytes incubated under the indicated experimental conditions. The graph shows mean normalized STIM1 band densities (± SEM, n = 4). Actin was used as a loading control. Open circles represent single determinations. **b** Upper panels, confocal microscopy images of cardiomyocytes under the experimental conditions indicated above the panels. Images show localization of STIM1 (red) with DAPI-labeled nuclei (blue). Lower panels are enlargements of the corresponding yellow boxed areas. **c** Representative western blots of Orai1 from cardiomyocytes incubated under the indicated experimental conditions. The graph shows mean normalized Orai1 band densities (± SEM, n = 4). Actin was used as a loading control. Open circles represent single determinations. **d** Confocal microscopy images of cardiomyocytes under the experimental conditions indicated above the panels. Images show localization of Orai1 (green) with DAPI-labeled nuclei (blue). Calibration bars, 10 μm in all cases. The graph shows mean numbers of particles (± SEM, n = 10) under the indicated experimental conditions. **e**, **f** Mean relative values (± SEM, n = 9–11) of mRNA expression from cardiomyocytes incubated in Dzx at the indicated times (filled bars). **p < 0.01, *p < 0.05

Dzx-induced increases in STIM1 and Orail (Fig. 3a, c) protein expression were attenuated by protein synthesis inhibition with CHX, although CHX did not alter STIM1 or Orail1 expression in cardiomyocytes not exposed to Dzx. Likewise, CHX blocked Dzx-induced changes in the distribution pattern of STIM1, but did not alter STIM1 distribution in cardiomyocytes not exposed to Dzx (Fig. 3b). Confocal microscopy analysis showed that CHX prevented the effects of opening mKATP channels on both Orai1 abundance and Orai1 particle counts (Fig. 3d).

To test whether upregulation of STIM1 and Orai1 proteins by Dzx is associated with the corresponding increases in transcription, the time course of expression levels of STIM1 and Orai1 mRNAs from cardiomyocytes incubated in Dzx was determined in qRT-PCR experiments. We observed significant increases in STIM1 and Orai1 mRNA levels after incubation with Dzx for 15 min (Fig. 3e, f). The overexpression of both transcripts was transient, with their levels decreasing to below control values by 60 min and 30 min after Dzx incubation onset, respectively.

### Upregulation of Orai1 and STIM1 and changes in distribution patterns involve the MAPK/ERK pathway and ROS

**Fig. 4:**
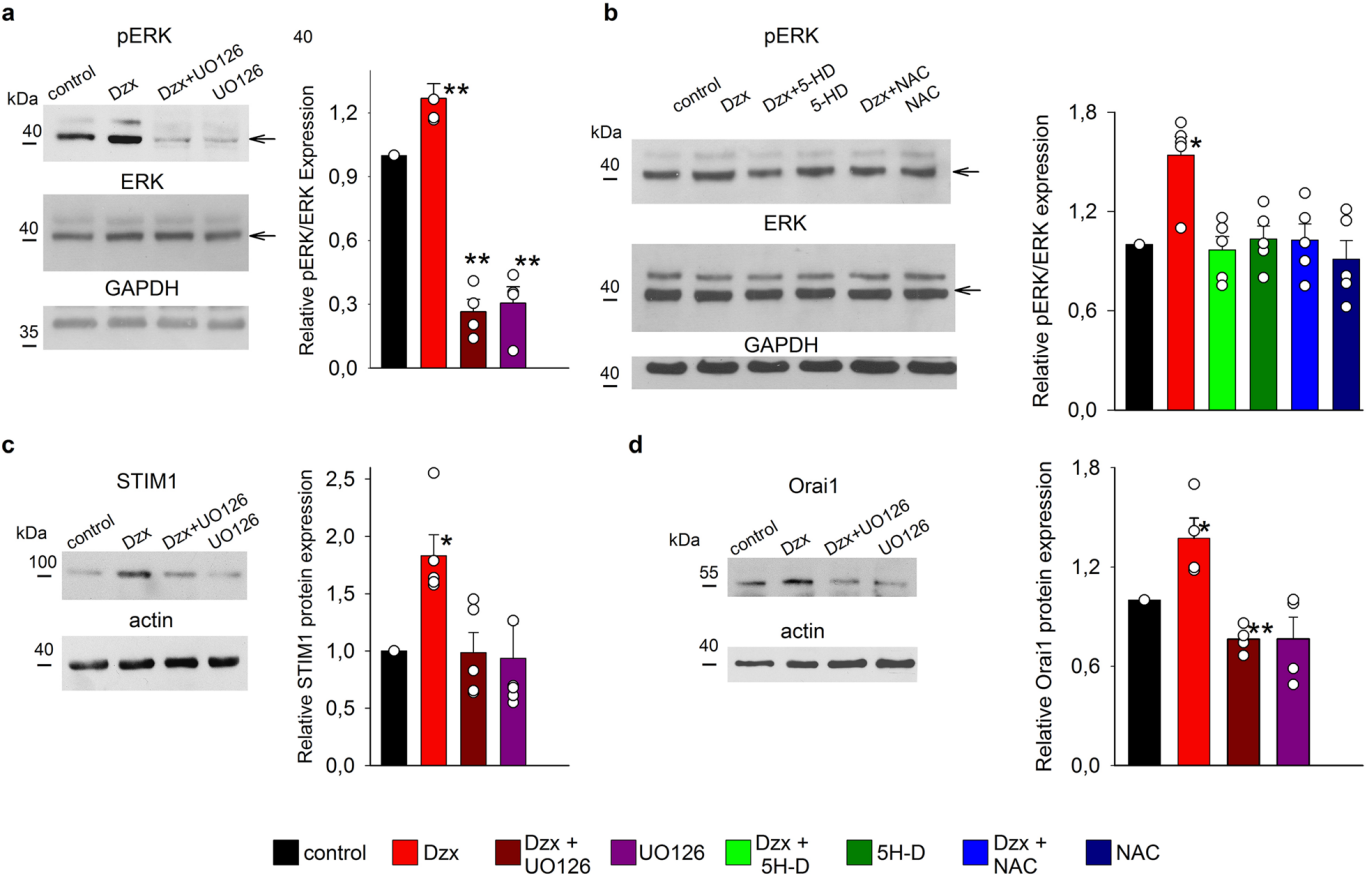
pERK is upregulated by Dzx treatment through ROS and is involved in upregulation of STIM1 and Orai1. **a** Upper panels, representative western blots of pERK from total extracts from isolated cardiomyocytes under the indicated experimental conditions. Middle panels, corresponding blots of total ERK. Lower panels, corresponding blots of GAPDH (loading control). The graph shows mean normalized pERK-band densities (± SEM, n = 4). Open circles represent single determinations. Arrows indicate the phosphorylated ERK (pERK, upper blot) and total ERK (ERK, middle blot) bands. **b** Representative western blots of pERK (upper blot) under the indicated experimental conditions. Middle panels, corresponding blots of total ERK. Lower panels, corresponding blots of GAPDH (loading control). The graph shows mean relative pERK expression levels (± SEM, n = 5). Open circles represent single determinations. **c** Representative western blots of STIM1 under the indicated experimental conditions. The graph shows mean STIM1 relative expression values (± SEM, n = 4). Open circles represent single determinations. **d** Representative western blots of Orai1 and actin under the indicated experimental conditions. The graph shows mean Orai1 relative expression values (± SEM, n = 4). Open circles represent single determinations. *p < 0.05, **p < 0.01

To test whether MAPK/ERK pathway signaling is involved in Dzx-induced upregulation of Orai1 and STIM1, we performed western blot experiments to detect possible changes in the abundance of phosphorylated ERK (pERK). The density of the pERK band increased in Dzx-treated cells and this effect was prevented in the presence of the MAPK/ERK inhibitor UO126, which by itself decreased pERK abundance below the control level. In contrast, the protein abundance of total ERK was unaffected by the treatments and the density of pERK bands was normalized relative to the density of ERK bands. GAPDH was used as loading control (Fig. 4a). To test whether phosphorylation of ERK is mediated by an increase in mitochondrial ROS production following the opening of mKATP channels, we measured pERK abundance in lysates from total fractions of cardiomyocytes preincubated with 5-HD or NAC. Western blots showed that incubation with Dzx increased pERK band density (Fig. 4a, b), and that this increase could be blocked by the presence of 5-HD or NAC, each of which did not significantly affect pERK expression in cells not exposed to Dzx. The protein abundance of total ERK was unaffected by the treatments and the density of pERK bands was normalized relative to the density of ERK bands. GAPDH was used as loading control (Fig. 4b).

**Fig. 5:**
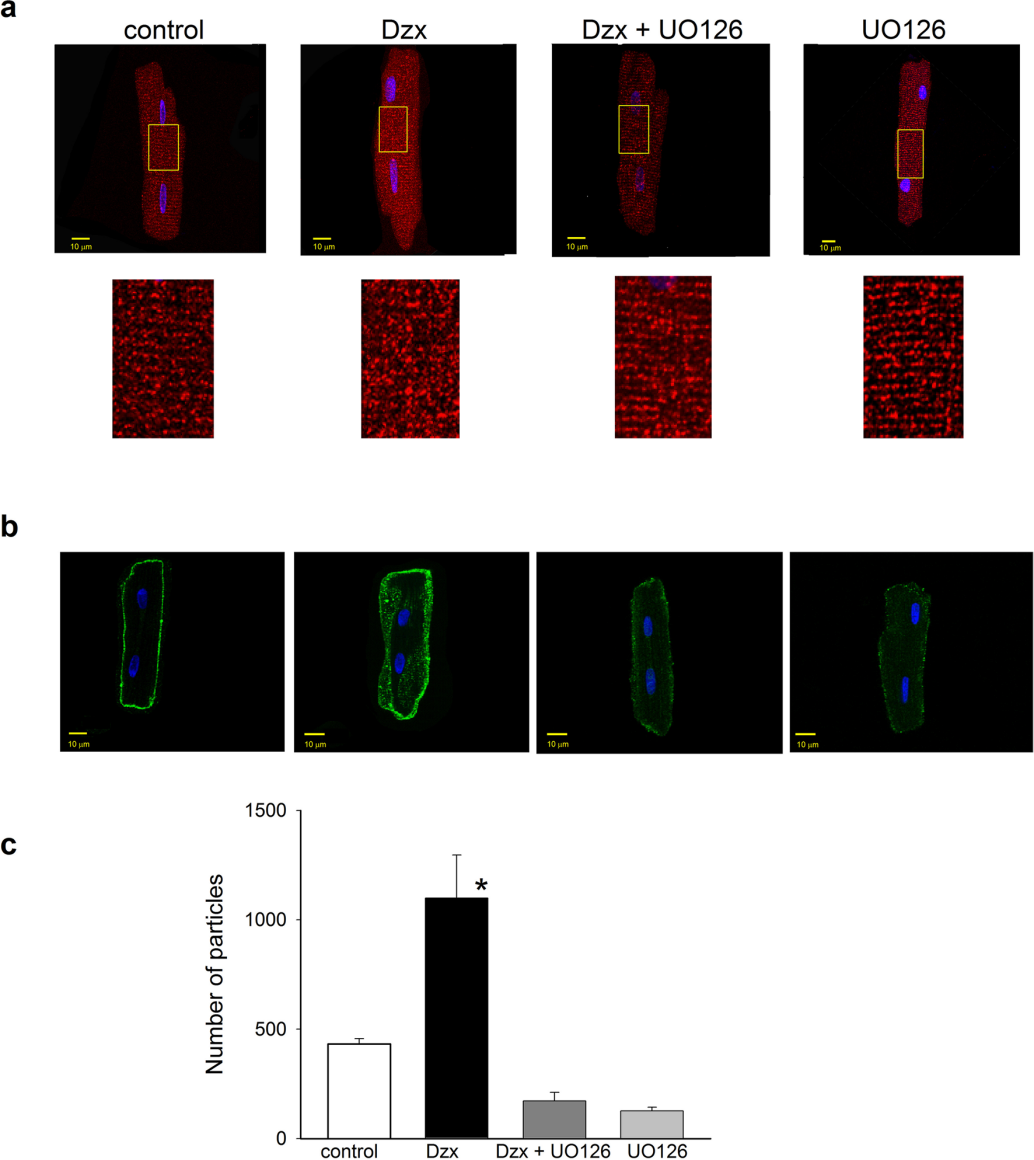
Role of MAPK/pERK signaling in Dzx-induced changes in STIM1 localization and upregulation of Orai1. **a** Upper panels, confocal microscopy images of cardiomyocytes under the indicated experimental conditions. Images show localization of STIM1 (red) with DAPI-labeled nuclei (blue). Lower panels, magnified views of the corresponding yellow boxed areas. **b** Representative confocal images of cardiomyocytes under the same conditions as in **a**. Images show localization of Orai1 (green) with DAPI-labeled nuclei (blue). **c** The graph shows mean Orail particle counts (± SEM, n = 5–6) from confocal images as in **b**. *p < 0.05

Increases in both STIM1 and Orail1 expression were also blocked by the MAPK/ERK inhibitor UO126 (Fig. 4c, d), whereas the inhibitor alone did not significantly affect STIM1 or Orail1 expression levels. Moreover, confocal microscopy experiments showed that UO126 suppressed Dzx-induced changes in the distribution pattern of STIM1 (Fig. 5a) as well as overexpression of Orai1 along the surface membrane of cardiomyocytes, as indexed by number of particles (Fig. 5b).

### Translocation of c-Fos and NF-κB by Dzx-treatment

**Fig. 6:**
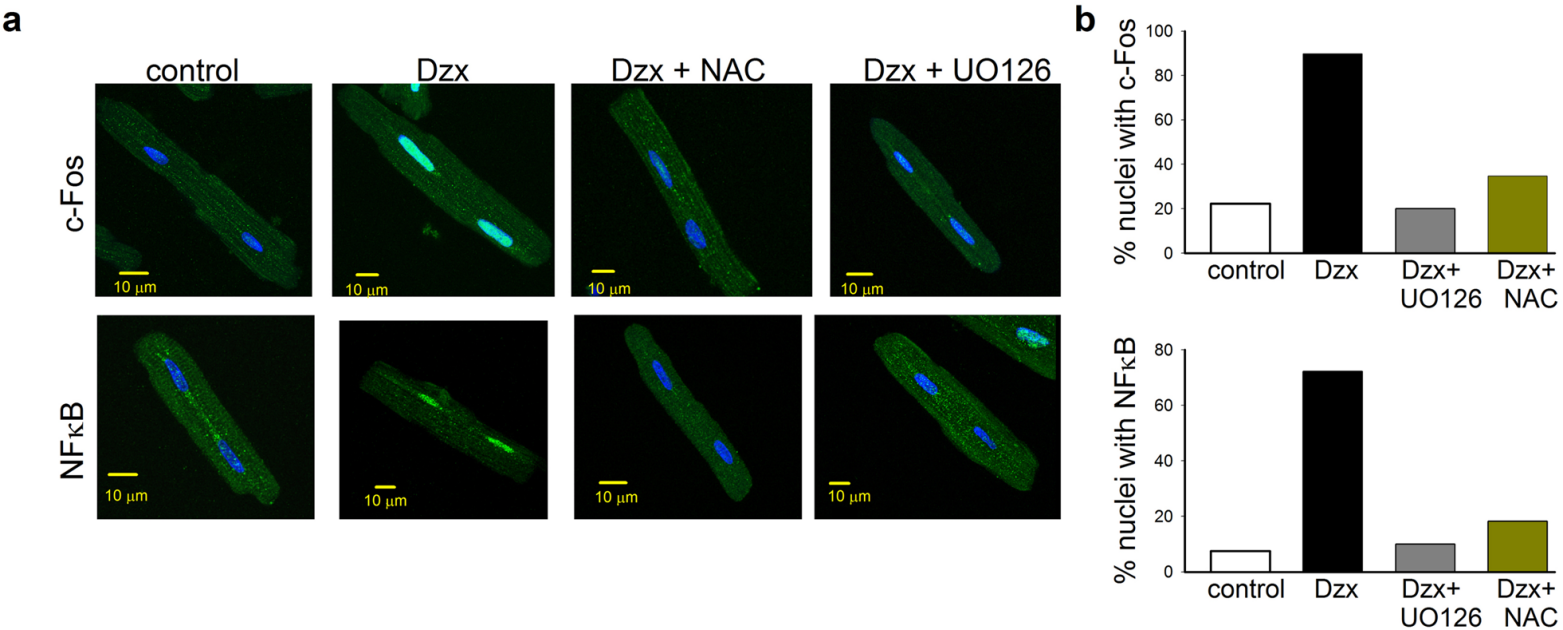
Involvement of ROS and the MAPK/pERK signaling in c-FOS and NFkB translocation in Dzx-treated cardiomyocytes. **a** Confocal microscopy images of cardiomyocytes under the experimental conditions indicated above the panels. Images show localization of c-FOS (green) or NFκB (green), with DAPI-labeled nuclei (blue). Calibration bars: 10 μm. **b** The graphs show the percentage of nuclei with c-Fos and NFκB under the indicated experimental conditions; 59 and 54 nuclei were considered, respectively

Confocal microscopy analysis showed that, after 15–30 min of Dzx incubation, both c-Fos and NF-κB had translocated to the nucleus (Fig. 6a, b). Note that the translocation of both transcription factors into the nucleus was blocked by NAC or UO126, consistent with the involvement of ROS and MAPK signaling in Dzx effects. Similar results were obtained in two independent experimental replicates.

## Discussion

In the present work, we presented the novel observation that both protein components of SOCE, STIM1 and Orai1, are upregulated by opening mKATP channels with Dzx. We also demonstrated for the first time in adult cardiomyocytes that STIM1 and Orai1 expression are upregulated through the MAPK pathway and that ROS are most likely involved. Upregulation of these two proteins was shown to be dependent upon de novo synthesis, as evidenced by the associated changes in the expression of their mRNA and protein abundance, with the protein synthesis inhibitor CHX preventing upregulation of STIM1 and Orai1.

In both ischemic preconditioning and preconditioning induced by drugs like Dzx, there are increases in the opening of mKATP channels and in ROS production [12, 13, 15, 21, 22] and in previous reports we showed that in cardiomyocytes, the increase in ROS production by Dzx is blocked by the ROS scavenger NAC [15, 16]. In present study, we used NAC and the mKATP channel blocker 5-HD to demonstrate involvement of ROS generated by mitochondria in upregulation of SOCE components. We also used H_2_O_2_ to assess the involvement of ROS directly and found that indeed incubation of H_2_O_2_ resulted in upregulation of STIM1.

Our experiments demonstrated nuclear translocation of NF-κB in Dzx-treated cardiomyocytes. Furthermore, our findings showing that NAC blocked both the translocation of this nuclear factor and upregulation of STIM1 and Orai1 suggest that these effects are related to ROS elevation. NFκB is a regulator of both STIM1 and Orai1 expression [23], and work done in mast cells and heterologous expression systems has demonstrated that binding of NFκB to specific sites within the promoter regions of the STIM1 and Orai1 genes increases the transcription of their mRNAs [24].

Although the targets of ROS remain to be identified, several possibilities may be suggested. ROS have been shown to activate SGK1 (serum and glucocorticoid regulated kinase 1) in non-excitable cells [24]. SGK1 is an enzyme that acts upstream of NFκB [25, 26] and is expressed in adult cardiomyocytes [27]. However, glucocorticoid stimulation decreases mRNA expression of Orai1 in rat cardiomyocytes although it increases SGK1-dependent SOCE activation [28]. Conversely, in the present experiments, we observed a ROS-dependent upregulation of Orai1 mRNA in Dzx-treated cardiomyocytes. Alternatively, it might be that ROS favor translocation of NFκB by oxidation-mediated activation [29] or via multiple ROS interactions in the NFκB signaling pathway. In many cases, ROS activate NFκB through the phosphorylation of proteins that bind NFκB [30]. Further research is needed to elucidate the detailed mechanism through which ROS may activate NFκB in Dzx-treated cardiomyocytes.

Our results suggest that upregulation of STIM1 and Orai1 by opening mKATP channels with Dzx involves ROS activation of the MAPK signaling pathway. ROS act as signaling molecules and have been shown to activate MAPKs in smooth muscle and other systems [31–34]. Involvement of ROS in MAPK activation in Dzx-treated cardiomyocytes is supported by several observations. Firstly, phosphorylation of ERK increased following Dzx-treatment, an effect that was blocked by 5-HD and NAC in the present study. Secondly, we found that Dzx-induced overexpression of STIM1 and Orai1 was blocked by UO126, a MAPK pathway inhibitor. Consistent with our observations, it has been reported that the opening of mKATP channels with Dzx increases phosphorylation of ERK in human monocytic cells [35]. Potentially, ROS could activate MAPK pathways by oxidative modifications of MAPK signaling proteins and/or by inactivation of MAPK phosphatases [34]. One of the earliest transcriptional events associated with MAPK pathway is activation of c-Fos, downstream of ERK [36]. In this study, we also observed translocation of c-Fos to the nucleus of Dzx-treated cardiomyocytes that was shown to be dependent on ROS and ERK by the actions of NAC and UO126, respectively. Involvement of ROS in c-Fos translocation is consistent with previous observations in cultured neonatal cardiomyocytes showing that H_2_O_2_ treatment can induce rapid increases in the expression of c-Fos [37]. Furthermore, the promoter region of the STIM gene has a putative c-Fos binding site [38]. STIM1 is upregulated in endothelial cells during sepsis, and its expression was shown to depend on cooperation between NFκB and p38 MAPK. Bacterial endotoxin induces time-dependent binding of NF-κB and c-Fos with sites in the STIM1 promoter region, and c-Fos silencing reduces STIM1 overexpression indicating that c-Fos can act as a potent regulator of STIM1 expression [38]. Hence, it is plausible that c-Fos may upregulate STIM1 in adult cardiomyocytes after Dzx treatment. Translocation of c-Fos and NFκB in Dzx-treated cardiomyocytes may activate genes other than those associated with STIM1 and Orai1. For example, in other systems activation of NfκB regulates the expression of hundreds of genes, some of them coding for stress response genes, while others are cell surface receptors and early response genes, among others (https://www.bu.edu/nf-kb/gene-resources/target-genes/). Further work is required to identify other possible targets of these transcription factors during pharmacological preconditioning in cardiomyocytes.

Upregulation of STIM1 and Orai1 is not necessarily accompanied by an increase in SOCE. SOCE depends on activation and Ca^2+^-dependent inactivation of Orai1 channels and previous work in HEK293 cells has shown that transfection of STIM1 and Orai1 lead to Ca^2+^ currents with more pronounced inactivation when the concentration of STIM1 plasmid increases and the concentration of Orai1 plasmid is kept constant [39]. Additionally, we have recently reported drastic decreases, rather than increases, in Ca^2+^ influx through store-operated Ca^2+^ channels in cardiomyocytes preconditioned with Dzx; these reductions in Ca^2+^ influx were related to increased ROS and Ca^2+^-dependent inactivation of Orai1 channels [16]. Our western blot experiments suggest an additional clue to decreased SOCE in spite of upregulation of STIM1 and Orai1. The stoichiometry between Orai1 and STIM1 is critical for SOCE-related channel function. In previously published experiments in which STIM1 and Orai1 were expressed at varying ratios in HEK cells, a highly non-linear, bell-shaped relationship between Orai1 expression and the amplitude of store-operated Ca^2+^ channel currents was seen, wherein the currents peaked at a ratio of about 2 STIM1 molecules per 1 Orai1 molecule [40], leading to the counterintuitive observation that increased Orai1 expression reduces SOCE [41]. This initially perplexing finding has been explained by an excess of Orai1 proteins competing for a limited number of STIM1 proteins. Our western blot experiments showed similar increases in STIM1 and Orail1 protein expression in Dzx-treated cardiomyocytes, which would thus be expected to lead to a deficit in the number of STIM1 proteins, and consequently deficient activation of Orai1 channels and therefore decreased SOCE.

Our immunofluorescence experiments suggest that additional factors may also contribute to SOCE reduction following myocytes incubation with Dzx. Notably, although Dzx increased STIM1 expression, it completely disrupted its striated distribution pattern in cardiomyocytes. Interestingly, the localization of STIM1 resembles the distribution of the junctional sarcoplasmic reticulum along the Z-disk [7]. We confirmed this distribution in control experiments wherein peaks of fluorescence were observed to be evenly distributed along the longitudinal axis, separated by distances consistent with sarcomere length. Moreover, in Dzx-treated cardiomyocytes, the number and size of particles associated with STIM1 fluorescence increased although the fluorescence peaks were no longer regularly spaced. Disruption in the localization of STIM1 by Dzx could result from increased expression of STIM1. However, transgenic mice with STIM1 overexpression in the heart showed partial co-localization of STIM1 with RyR2 [3], a distribution pattern completely different from the one we observed by Dzx treatment, Therefore, it is likely that the lack of regularly spaced fluorescence peaks observed after incubation in Dxz does not originate by STIM1 overexpression per se and it is expected to further contribute to reduced SOCE during pharmacological preconditioning with Dzx. In short, we propose that changes in the STIM1/Orai1 ratio and disruption in the distribution pattern of STIM1 contribute to reduced SOCE during pharmacological preconditioning.

A reduction in SOCE is likely beneficial under conditions of ischemic stress. Ischemic preconditioning and pharmacological preconditioning with openers of mKATP channels like Dzx involve changes in Ca^2+^ homeostasis that can mitigate the effects of drastic increases in cytosolic Ca^2+^ caused by ischemia [15, 42]. The sarcoplasmic reticulum Ca^2+^ content is markedly depleted in intact hearts subjected to ischemia and reperfusion [43], which would be expected to be followed by activation of SOCE. A decrease in SOCE following Dzx-induced preconditioning would therefore moderate the drastic increase in cytosolic Ca^2+^ caused by ischemia that is associated with cell death, thereby resulting in protection from an ischemic insult. Consistent with this possibility, the SOC inhibitor glucosamine [44–46] has been shown to provide protection against I/R injury [47], suggesting that SOCE plays a role in Ca^2+^ overload [46].

## Conclusion

In conclusion, the present results suggest that opening mKATP channels with Dzx produces de novo synthesis of STIM1 and Orai1 by a mechanism that involves NFkB, c-Fos, and ROS via MAPK/ERK signaling. Upregulation of SOCE components and disruption in the distribution pattern of STIM1 by Dzx may contribute to decrease Ca^2+^ influx in cardiomyocytes under stress conditions and to cardioprotection against ischemic insults.

## Supplementary information

**Additional file 1: Figure S1.** Cardiomyocyte protein abundance of STIM1 is increased by ROS. **a** A representative STIM1 western blot of whole membrane fractions from control and H_2_O_2_ (100 μM)-treated cardiomyocytes with GAPDH as a loading control. **b**, Mean relative STIM1 abundance values under control conditions and after application of H_2_O_2_. Open circles represent single determinations. Values are expressed as mean ± SEM, n = 3. *p < 0.05.

## Data Availability

The data and materials used and/or analyzed during the current study are available from the corresponding author on reasonable request.
