# Supplementary figures and images for: The mitochondrial K-ATP channel opener diazoxide upregulates STIM1 and Orai1 via ROS and the MAPK pathway in adult rat cardiomyocytes
Source: Cell Biosci. 2020 Aug 13;10:96. doi: 10.1186/s13578-020-00460-w (PMC7424994; doi:10.1186/s13578-020-00460-w)

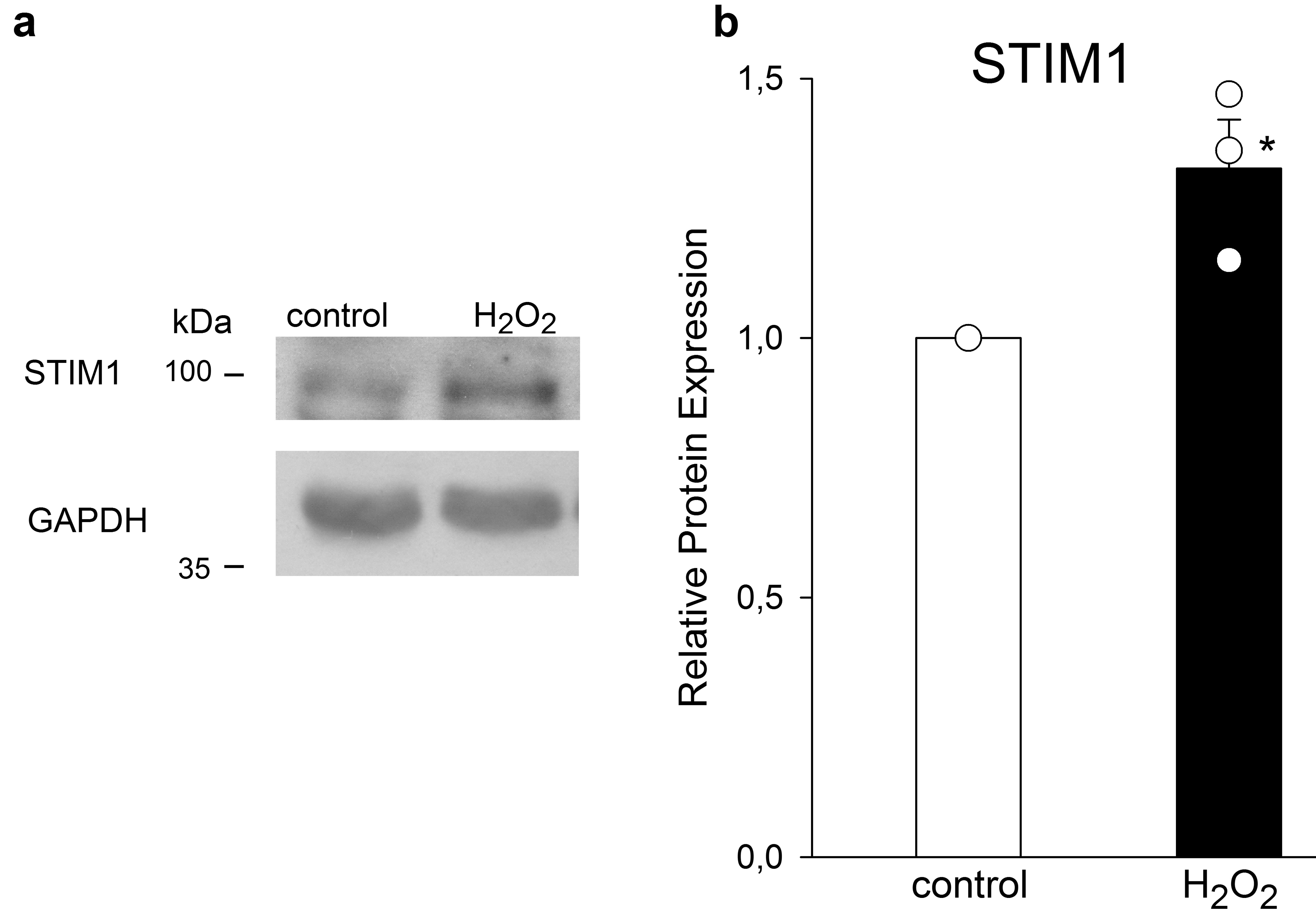

Supplement: Supplementary file 1 — Additional file 1: Figure S1. Cardiomyocyte protein abundance of STIM1 is increased by ROS. a A representative STIM1 western blot of whole membrane fractions from control and H2O2 (100 μM)-treated cardiomyocytes with GAPDH as a loading control. b, Mean relative STIM1 abundance values under control conditions and after application of H2O2. Open circles represent single determinations. Values are expressed as mean ± SEM, n = 3. *p < 0.05. [file 13578_2020_460_MOESM1_ESM.jpg]
